# Electroacupuncture Treatment Normalized Sleep Disturbance in Morphine Withdrawal Rats

**DOI:** 10.1093/ecam/nep133

**Published:** 2011-05-26

**Authors:** Yi-Jing Li, Fei Zhong, Peng Yu, Ji-Sheng Han, Cai-Lian Cui, Liu-Zhen Wu

**Affiliations:** Neuroscience Research Institute, Department of Neurobiology, School of Basic Medical Sciences, Peking University, Key Lab for Neuroscience, the Ministry of Education and Key Lab for Neuroscience, the Ministry of Public Health, Beijing 10019 1, China

## Abstract

Sleep disturbance is considered as an important symptom of acute and protracted opiate withdrawal. Current results suggest that sleep disturbance may be taken as a predictor of relapse. Appropriate sleep enhancement therapy will be in favor of the retention in treatment for opiate addicts. Our previous studies have shown that electroacupuncture (EA) is effective in suppressing morphine withdrawal syndrome. The aim of the present study is to investigate the effect of 2 and 100 Hz EA on the sleep disturbance during morphine withdrawal. Rats were made dependent on morphine by repeated morphine injections (escalating doses of 5–80 mg kg^−1^, subcutaneously, twice a day) for 5 days. EA of 2 or 100 Hz was given twice a day for 3 days, starting at 48 h after the last morphine injection. Electroencephalogram and electromyogram were monitored at the end of the first and the last EA treatments, respectively. Results showed that non-rapid eye movement (NREM) sleep, REM sleep and total sleep time decreased dramatically, while the sleep latency prolonged significantly during acute morphine withdrawal. Both 2 and 100 Hz EA produced a significant increase in NREM sleep, REM sleep and total sleep time. It was suggested that EA could be a potential treatment for sleep disturbance during morphine withdrawal.

## 1. Introduction

Sleep disturbance is a significant feature of acute and protracted withdrawal syndrome during detoxification from opiates. Howe et al. [1, 2] reported that heroin-dependent patients showed a marked increase in waking and decrease in both slow wave and rapid eye movement (REM) sleep during acute heroin withdrawal. The patients' total sleep time was dramatically suppressed on Days 2 and 3 of withdrawal, and remained below normal control values on Days 5–7.

Methadone, as a long-acting opioid-agonist, is widely used as a substitution of heroin for the treatment of withdrawal syndrome and prevention of relapse in heroin-dependent patients. However, the opiate addicts often complain of sleep difficulties during methadone maintenance treatment (MMT). Some of them, in fact, attribute their premature exit from this therapy to sleep difficulties [3–5]. Indeed, the persistent insomnia in drug-dependency may provoke heroin relapse during methadone maintenance [6].

Our previous clinical studies have demonstrated that transcutaneous electrical acupoint stimulation (TEAS) could ameliorate heroin abstinence syndrome and postpone relapse in heroin addicts [7, 8]. In fact, TEAS was reported to show an immediate hypnotic effect during the treatment in one-third of the patients [9]. It seemed rational to further study the effect of electroacupuncture (EA) on sleep enhancement with measurement of electroencephalogram (EEG) and electromyogram (EMG) in animal model of morphine withdrawal.

The purpose of this study was to characterize the sleep profile of morphine withdrawal rats with EEG and EMG, and to evaluate whether 2 and 100 Hz EA can improve sleep disturbance during morphine withdrawal.

## 2. Methods

### 2.1. Animals

The animals used were male Sprague-Dawley rats (Grade 1, purchased from Animal Center of Peking University, Beijing), weighing 220–250 g each. Each rat was housed individually in a thermoregulated room (22–24°C) with a 12 : 12 h light-dark cycle, and had access to food and water *ad libitum*. All experimental procedures were approved by the Animal Use Committee of Peking University Health Science Center and carefully designed to minimize the number of animals used and their suffering.

### 2.2. Fixation of Electrodes

Sterile surgery was performed under general anesthesia induced by sodium pentobarbital (40–50 mg kg^−1^, intraperitoneally). The rats were placed in a Kopf stereotaxic instrument with blunt ear bars. Two stainless-steel screws attached to insulated wire were implanted on the skull over the frontal-parietal cortex to record the EEG. Two electrodes were placed 2 mm anterior and 5 mm lateral to the bregma. Two insulated stainless-steel wires bared at the tips were sutured onto the dorsal cervical neck muscles to record the EMG. All of these electrodes were attached to a miniature connector. The implant assembly was affixed to the skull with dental acrylic [10]. All animals were allowed 7 days for recovery before the onset of experiments.

### 2.3. Recording Procedures and Computerized Analysis of EEG and EMG Signals

**Figure 1:**
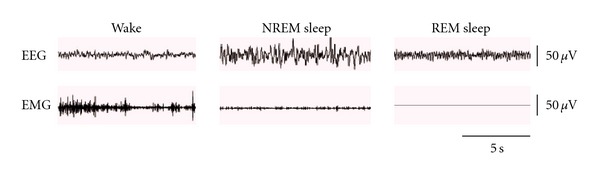
Typical samples of EEG and EMG classified as wake, NREM sleep and REM sleep.

For electrophysiological recording, a lightweight shielded cable which was attached to a counterbalanced swivel permitting free movement was connected to the plug on the rat's head. All rats were studied in their home-cages in a noise-attenuated environment, free from any interruption. Cortical EEG and EMG signals were amplified and filtered (EEG, 0.5–30 Hz; EMG, 20–200 Hz), then digitized at a sampling rate of 200 Hz and recorded by biopac MP 150 system (Santa Barbara, CA, USA). After that, polygraphic recordings were automatically scored offline by 4-s epochs as wakefulness, non-REM (NREM) sleep and REM sleep by SleepSign software (Kissei Comtec, Nagano, Japan). Initially, an expert observer selected the characteristic samples from the three stages of vigilance (wake, NREM and REM sleep) in each day recording of each rat, according to previously validated criteria [10–12] as follows: (i) wakefulness was identified by low-amplitude, fast EEG activity (mainly in *α* (8–13 Hz) and *β* (13–30 Hz) band) and high EMG activity; (ii) NREM sleep was identified by high-amplitude, slow EEG activity (delta activity, 0.5–4 Hz, was increased to dominate the power spectra) and low EMG activity relative to that of waking. NREM sleep was considered initiated if six consecutive 4-s epochs (24 s) were scored as NREM sleep; (iii) REM sleep was identified by the presence of low-amplitude and regular theta activity (4–8 Hz) on EEG, together with very low EMG activity (shown in Figure 1). The criteria of the auto-scoring could then be configured and saved in SleepSign software according to these reference samples. As a final step, defined sleep-wake architectures were examined visually and corrected if necessary.

### 2.4. Drugs and Drug Administration

**Figure 2:**
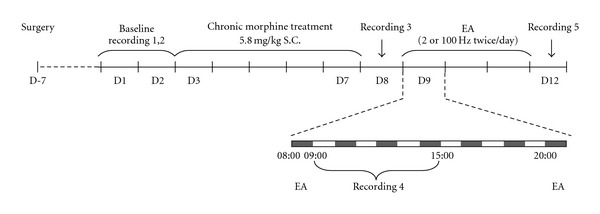
Schematic diagram for experimental design.

The rats were injected subcutaneously with morphine hydrochloride in normal saline twice a day (at 8:00 and 20:00 h) at the following doses per injection: 5 mg kg^−1^ on the first day, followed by 10, 20, 40, 80 mg kg^−1^, respectively. The first injection (5 mg kg^−1^) was on the night of Day 2 (shown in Figure 2), while the last injection (80 mg kg^−1^) was given on the morning of Day 7. The injection volume was 2 ml kg^−1^.

### 2.5. EA

Rats were kept in specially designed holders, with their hind legs and tails exposed. Two stainless-steel needles of 0.25 mm diameter and 5 mm in length were inserted into two sites on the hind legs: one is the “Zusanli” point (ST36) near the knee joint (5 mm lateral to the anterior tubercle of tibia) and the other is the “Sanyinjiao” point (SP6) near ankle joint (at the level of the superior border of the medial melleolus, between the posterior border of the tibia and the anterior border of the Achilles tendon). Constant current square-wave electrical stimulation produced by a programmed pulse generator HANS LH-800 (Beijing Astronautics and Aeronautics Aviation University, Beijing, China) was given for a total of 30 min. The frequency of stimulation used was 2 Hz (0.6 ms pulse width) or 100 Hz (0.2 ms pulse width). The intensity of the stimulation was increased stepwise from 0.5 to 1 mA, and ended at 1.5 mA, with each step lasting for 10 min.

### 2.6. Experimental Procedures

As shown in Figure 2, after 7-day recovery from surgery, all rats received 2-day recording of the EEG and EMG, starting at 9:00 AM and lasting for 6 h per day (baseline recordings 1 and 2), the mean values of NREM, REM and total sleep time were served as baseline. All rats were then administered subcutaneously with escalating dose of morphine hydrochloride. Twenty-four hours after the last injection of morphine, sleep-wakefulness state was monitored for 6 h (recording 3) to assess the effect of morphine withdrawal on the sleep state.

Forty-eight hours after the last injection of morphine, rats were randomly divided into three groups of five each. Group 1 received no treatment serving as blank control group, groups 2 and 3 were given 2 and 100 Hz EA, respectively twice a day for 3 days. After the first EA treatment (Day 9 morning, 30 min after the first EA treatment) and 3-day EA treatment (Day 12 morning), all rats received two recordings of the EEG and EMG (recordings 4 and 5) to observe the effect of single or multiple EA treatment on sleep state in morphine withdrawal rats.

### 2.7. Data Analysis

Data were processed using commercially available software GraphPad PRISM 4.0. Data were presented as mean  ±  standard error of the mean (SEM) and analyzed with two-way ANOVA followed by Bonferroni post-test. Moreover, two-tailed paired *t*-test was used where appropriate, with each animal serving as its own control. Statistical significance was set at *P* < .05.

## 3. Results

### 3.1. Acute Withdrawal from Chronic Morphine Treatment Caused Sleep Disturbance

**Figure 3:**
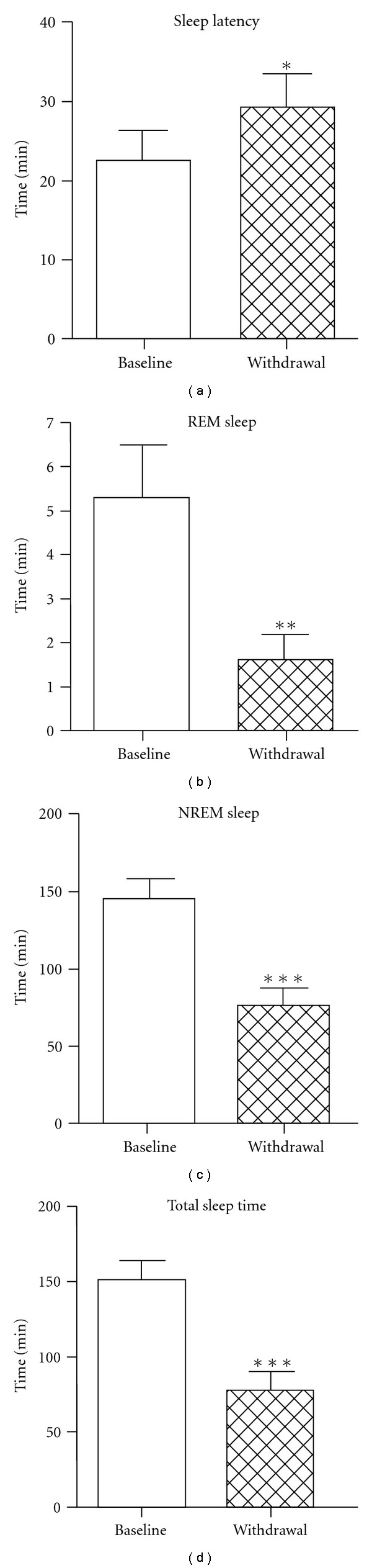
Sleep latency, time spent in NREM, REM sleep and total sleep time during 6-h recording in baseline and acute withdrawal days. Empty and filled columns show the data in baseline day and acute withdrawal day (starting at 24 h after last morphine injection), respectively. *P < .05; **P < .01; ***P < .001 compared with baseline by paired *t*-test; *n* = 15.

Comparison of classical sleep parameters (shown in Figure 3 and Table 1, sleep latency; REM sleep; NREM sleep; total sleep time) between baseline days (recordings 1 and 2) and acute withdrawal day (recording 3, starting at 24 h after last morphine injection) by two-tailed paired *t*-test revealed a significant decrease in NREM sleep (47.57% reduction), REM sleep (69.53% reduction) and total sleep time (48.34% reduction). Meanwhile, the sleep latency was significantly longer (29.75% increase) during acute withdrawal.

### 3.2. Single 100 Hz EA was Better than 2 Hz on Increasing Sleep Time in Morphine Withdrawal Rats

After recording of the sleep state on the first withdrawal day, rats were randomly divided into three groups of five each. EA of 2 or 100 Hz was given for 3 days twice a day from 48 h after last morphine injection (Day 9). Rats of the control group were staying in home-cage. In order to investigate the effect of single EA treatment on sleep disturbance, EEG and EMG of all three groups were monitored for 6 h (recording 4) at the end of the first EA treatment. Results were defined as percent difference of sleep time between the experiment day and the baseline day over baseline (% (sleep time in experiment day—sleep time in baseline day)/baseline).

**Figure 4:**
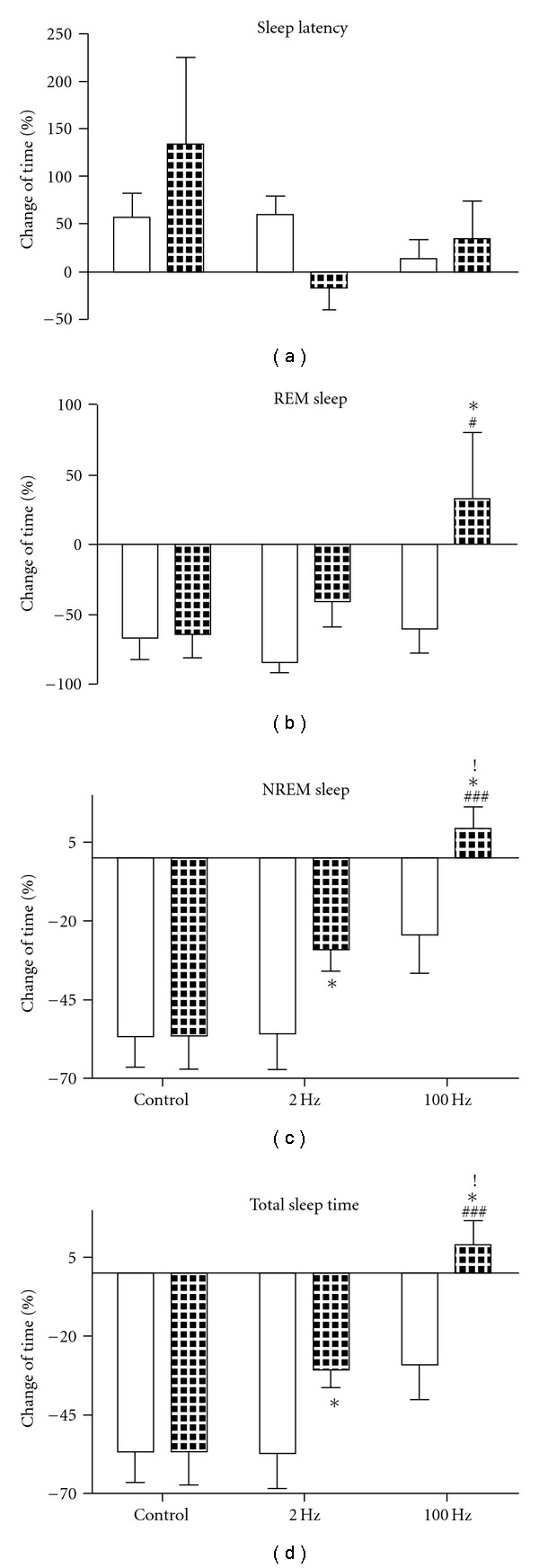
Effect of single EA treatment on sleep latency (a), time spent in NREM (c), REM sleep (b) and total sleep time (d) during 6-h recording after morphine withdrawal. Empty columns show the data during acute morphine withdrawal before various treatments, and filled columns after the treatment (single session of 2 Hz, 100 Hz or without EA in control group). *P < .05 compared with the data before treatment; ^#^ *P* < .05, ^###^ *P* < .001 compared with data after treatment in control group. ^!^ *P* < .05 compared with the data after treatment in 2 Hz group. *n* = 5.

Figure 4(a) illustrates that single EA treatment (2 or 100 Hz) produced no significant change of the sleep latency. Two-way ANOVA indicated no significant interaction between treatment and time (*F*_2, 24_ = 1.49; *P* = .2465), nor any significant difference between pre- and post-treatment (*F*_1, 24_ = 0.04; *P* = .8384) and among the groups of animals that received different treatments (no EA, 2 or 100 Hz EA) (*F*_2, 24_ = 1.75; *P* = .1954).

However, single session of EA did improve the sleep profile depicted by sleep time.

Figure 4(b) shows the effect of single EA treatment on REM sleep. Two-way ANOVA indicated significant difference between pre- and post-treatment (*F*_1, 24_ = 5.45; *P* = .0282). Single 100 Hz EA significantly increased the REM sleep (compared with control group and pre-treatment by Bonferroni post-test).

Figure 4(c) shows the effect of single EA treatment on NREM sleep. Two-way ANOVA for treatment × time showed significant effects for treatment (*F*_2, 24_ = 12.46; *P* = .0002) and time (*F*_1, 24_ = 6.02; *P* = .0218), with no interaction (*F*_2, 24_ = 1.56; *P* = .2310). After the single session of either 2 or 100 Hz EA, time spent in NREM sleep was raised apparently (100 Hz group: compared with control group and pre-treatment by Bonferroni post-test; 2 Hz group: compared with pre-treatment by paired *t*-test). The 100 Hz group showed better effect than 2 Hz (compared with data after 2 Hz EA by Bonferroni post-test).

Figure 4(d) shows that either 2 or 100 Hz EA increased the total sleep time (100 Hz group: compared with control group and pre-treatment by Bonferroni post-test; 2 Hz group: compared with pre-treatment by paired *t*-test). Two-way ANOVA indicated significant effects for treatment (*F*_2, 24_ = 12.98; *P* = .0002) and time (*F*_1, 24_ = 7.74; *P* = .0103), with no interaction (*F*_2, 24_ = 2.11; *P* = .1426). The effect of 100 Hz EA was better than 2 Hz (compared with data after 2 Hz by Bonferroni post-test).

### 3.3. Multiple Application of 2 or 100 Hz EA was Both Effective in Normalizing Sleep Time in Morphine Withdrawal Rats

When 3-day EA treatment was finished on the night of Day 11, all rats received another recording of EEG and EMG (on Day 12 morning, recording 5) to assess the effect of multiple application of EA on sleep disturbance.

**Figure 5:**
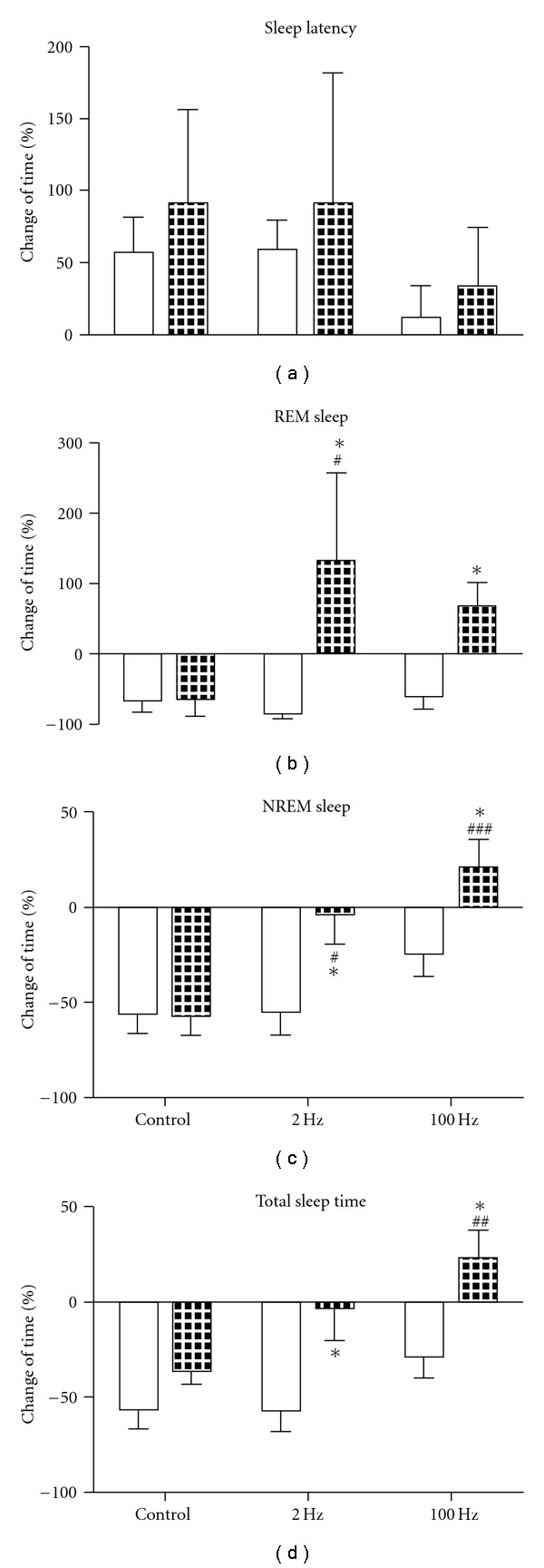
Effect of 3-day EA treatment on sleep latency (a), time spent in NREM (c), REM sleep (b) and total sleep time (d) during 6-h recording after morphine withdrawal. Empty columns show the data during acute morphine withdrawal before various treatments and filled columns after the treatment (3-day EA treatment of 2 Hz, 100 Hz or without EA in the control group). *P < .05 compared with the data before treatment; ^#^ *P* < .05, ^##^ *P* < .01, ^###^ *P* < .001 compared with data after treatment in control group. *n* = 5.

After 5-day withdrawal from chronic morphine injection, the REM sleep, NREM sleep and total sleep time were still below the baseline values in the control group, with longer sleep latency (Figure 5).

Figure 5(a) shows that for sleep latency, no statistical difference was found among all the groups.

Figure 5(b) displays that multiple application of either 2 or 100 Hz EA markedly increased REM sleep compared with pretreatment level. There was a significant effect for time (*F*_1, 24_ = 6.68; *P* = .0163).

Figure 5(c) shows the effect of multiple EA treatment on NREM sleep. Two-way ANOVA for treatment × time showed significant effects for treatment (*F*_2, 24_ = 9.13; *P* = .0011) and time (*F*_1, 24_ = 9.59; *P* = .0049), with no interaction (*F*_2, 24_ = 2.42; *P* = .1102).

Figure 5(d) shows the effect of multiple EA treatment on total sleep time. Two-way ANOVA indicated significant effects for treatment (*F*_2, 24_ = 6.38; *P* = .0080) and time (*F*_1, 24_ = 17.36; *P* = .0003), with no interaction (*F*_2, 24_ = 1.2; *P* = .3186).

*Post hoc* analysis showed that after multiple application of either 2 or 100 Hz EA, REM, NREM sleep and total sleep time were obviously increased (compared with control group and pre-treatment level by Bonferroni post-test. REM sleep after 100 Hz treatment was compared with pre-treatment level by paired *t*-test).

## 4. Discussion

During acute abstinence, there was a dramatic decrease of REM, NREM sleep and total sleep time, with prolonged sleep latency in morphine withdrawal rats. The sleep disturbance remained for at least 5 days after the last morphine injection. This was similar to reports in opiate addicts and findings of other investigators who used different procedure to produce morphine dependence [1, 2, 13]. Therefore, the animal model adopted here appeared to be suitable for studying sleep impairment during morphine withdrawal.

The mechanism of the sleep disturbance during withdrawal remains unclear.

Wake-sleep regulatory system includes the arousal-circuitry which promotes wakefulness and the sleep-producing circuitry. Mutual inhibition between these two neural circuits results in switching properties that define discrete wake and sleep states [14].

One important branch of the ascending arousal system originates from monoaminergic neurons in the upper brainstem and caudal hypothalamus, including the locus coeruleus (LC), dorsal and median raphe nuclei, ventral periaqueductal gray matter and tuberomammillary neurons. Their projections activate neurons throughout the cerebral cortex. In addition, the input to cerebral cortex is augmented by lateral hypothalamic peptidergic neurons and basal forebrain neurons. Firing of the monoaminergic neurons usually increase during wakefulness, slow down during NREM sleep and stop altogether during REM sleep [14].

Opioid receptors have been found in the brain regions that are involved in sleep regulation and it has been suggested that endogenous opioids play a role in the induction and maintenance of sleep state [12, 15–17].

Enkephalin containing neurons are widely distributed in the brain region related to sleep regulation such as the solitary tract nucleus, the preoptic area and the raphe. Enkephalinergic fibers projecting to the noradrenergic LC neurons exert an inhibitory effect on the activity of postsynaptic neurons by locally delivered opioids, and result in decreased wakefulness and increased slow wave sleep [17].

Dynorphin inhibits noradrenergic LC neurons by suppressing excitatory inputs via presynaptic kappa-receptors [18].

Exogenous opioids such as morphine bind to the same site as the endogenous opioids and suppress the production of endogenous opioids, thereby reduce their control on sleep [17]. When the morphine exposure is stopped abruptly, the functions of endogenous opioids are still below the normal level [19, 20], which may cause a rebound of the function of LC or other wake-sleep regulatory system in morphine withdrawal rats. Indeed, evidence obtained from electrophysiological studies revealed the hyperactivity of LC during morphine withdrawal [15, 21], which will lead to an elevated release of noradrenaline [22, 23]. In this regard, one could expect that the sleep disturbance during morphine withdrawal is due, at least in part, to the decreased endogenous opioids level and the resultant disinhibition of noradrenergic neurons in the LC.

In addition to distorted noradrenergic neurotransmission, changes in glutamatergic, GABAergic, serotonergic and cholinergic systems during morphine withdrawal [24–31] have also been shown to play important roles in sleep homeostasis [32].

It is worth noting that the neuropeptide orexin in the lateral hypothalamus is reported to play a role in manipulating sleep-waking cycle and responses to morphine [33–35]. Morphine withdrawal produces acute activation of orexin cells accompanied by an increase of orexin gene expression. On the contrary, orexin knock-out mice display attenuated morphine withdrawal syndrome [33, 34].

In the present study, we found that single session of EA alleviated the sleep disorder in morphine withdrawal rats, with 100 Hz more effective than 2 Hz. After 3-day EA treatment, all three parameters of sleep time (REM, NREM and total sleep time) returned to baseline level. In this case, 2 Hz was as effective as 100 Hz.

Our previous works showed that 2 Hz EA produced a significant increase in the content of enkephalin and *β*-endorphin, while 100 Hz increased that of dynorphin in central nervous system [36]. We also reported that 100 Hz EA increased the spinal dynorphin release in morphine-dependent rats after withdrawal, an effect readily reversed by intrathecal injection of dynorphin antiserum or kappa opioid antagonist nor-BNI. These results suggested that dynorphin and kappa opioid receptors might be responsible for ameliorating withdrawal syndrome [20].

We presumed that the facilitated release of endogenous opioids by 2 and 100 Hz EA probably compensated the deficiency of the endogenous opioids during morphine withdrawal, thereby inhibited the hyperactivity of LC neurons and restored the sleep-wake homeostasis.

Dynorphin may also participate in the sleep regulation by exerting influences on sleep-promoting neurons. It is well known that there is a population of sleep-promoting neurons in the ventrolateral preoptic nucleus (VLPO), which is connected with ascending arousal system mentioned earlier [37]. Lesion of VLPO by ibotenic acid resulted in loss of NREM and REM sleep [38]. Recent study demonstrated that kappa opioid receptors were expressed in VLPO neurons, and direct infusion of dynorphin A into VLPO increased NREM sleep [12]. Therefore, VLPO might be another important brain target where 100 Hz EA-accelerated release of dynorphin plays a role in improving the insomnia during abstinence.

Earlier works of Guo et al. [39–41] reported that 2 Hz EA increased preproenkephalin (PPE) mRNA level, whereas 100 Hz EA increased preprodynorphin (PPD) mRNA level in brain, which might account for the cumulative therapeutic effect on sleep disturbance with 3-day EA treatment.

More information is needed to fully understand the mechanisms underlying sleep disturbance during morphine withdrawal and its modulation by EA.

Opiate dependence is characterized by a high incidence of relapse following detoxification [42]. Sleep disturbance is positively correlated with increased potential of relapse to heroin abuse. Individuals with shorter sleep time seem more likely to leave treatment earlier [43]. If the problem of sleep disturbance is resolved, the risk of relapse may be reduced. Therefore, the improvement of sleep profile during morphine withdrawal by 2 and 100 Hz EA may provide useful information for the design of appropriate care for opiate addicts in drug abstinence.

## Funding

National Basic Research Program grant (2009CB522003); National Natural Science Foundation grant (30770690) of China.

## Figures and Tables

**Table 1:** Changes of sleep time and sleep latency in minutes in acute morphine withdrawal rats.

	Baseline (B)	Withdrawal (W)	W/B (%)	*P-*value
NREM sleep	145.6 ± 12.93	76.34 ± 11.74	52.43	.0001
REM sleep	5.284 ± 1.192	1.610 ± 0.5483	30.47	.0030
Total sleep time	150.9 ± 13.25	77.95 ± 11.98	51.66	.0001
Sleep latency	22.59 ± 3.759	29.31 ± 4.305	129.75	.0260

All the values in this table are min  ±  SEM.
